# ARID1A Downregulation Predicts High PD-L1 Expression and Worse Clinical Outcome in Patients With Gallbladder Cancer

**DOI:** 10.3389/fonc.2022.787897

**Published:** 2022-02-07

**Authors:** Lingxi Nan, Changcheng Wang, Jie Wang, Shulong Zhang, Xiaobo Bo, Yueqi Wang, Houbao Liu

**Affiliations:** ^1^ Department of General Surgery, Zhongshan Hospital, Fudan University, Shanghai, China; ^2^ Biliary Tract Diseases Institute, Fudan University, Shanghai, China; ^3^ Shanghai Engineering Research Center of Biliary Tract Minimal Invasive Surgery and Materials, Shanghai, China; ^4^ Department of Thoracic Surgery, Fudan University Shanghai Cancer Center, Shanghai, China; ^5^ Department of General Surgery, Xuhui District Central Hospital, Shanghai, China; ^6^ Cancer Center, Zhongshan Hospital, Fudan University, Shanghai, China

**Keywords:** ARID1A, gallbladder cancer, PD-L1, PD1, prognosis, tumor immune

## Abstract

**Background:**

Recent studies have confirmed that AT-rich interactive domain-containing protein 1A (ARID1A) plays a critical role in tumorigenesis, but its role in gallbladder cancer (GBC) remains unclear.

**Methods:**

In total, 224 patients from Zhongshan Hospital were recruited for this retrospective study. The clinicopathological and baseline characteristics of the patients were collected. Bioinformatics analysis was performed to reveal variations in genes and signaling pathways, and ARID1A and PD-L1 expression and the number of PD1+ tumor-infiltrating lymphocytes (TILs) were measured by immunohistochemical staining.

**Results:**

ARID1A expression was negatively correlated with overall survival in patients with GBC, and multivariate analysis identified ARID1A as an independent prognostic factor for overall survival. A heatmap and gene set enrichment analysis suggested that cytotoxic T lymphocyte signatures and immune-related signaling pathways were downregulated in ARID1A low tumors. Subsequent immunohistochemical staining confirmed that ARID1A expression was negatively correlated with PD-L1 expression and PD1+ TILs in the tumor microenvironment. The Kaplan–Meier analysis suggested that high ARID1A expression combined with low PD-L1 expression or low PD1+ TIL counts is associated with the best prognosis in patients with GBC.

**Conclusion:**

ARID1A inactivation can lead to a worse prognosis in patients with GBC, potentially by mediating immune evasion through the PD1/PD-L1 pathway.

## Introduction

Gallbladder cancer (GBC) is a rare malignant tumor of the digestive tract and the most common malignancy of the biliary tract system. Most patients with GBC already have advanced disease at diagnosis, and the 5-year survival rate is less than 5% (1). At present, the prognosis of patients with GBC is mainly predicted according to the clinical and pathological stages, such as TNM and the American Joint Committee on Cancer (AJCC) stage (2). However, the lack of tumor markers represents a significant deficiency of these staging systems. There is thus an urgent need to identify novel biomarkers to facilitate the prediction of prognosis in GBC.

The switch/sucrose non-fermentable (SWI/SNF) chromatin remodeling protein complex, which consists of 12–15 subunits, is assembled from the products of 29 genes (3). The SWI/SNF complex can alter the structure of chromatin and regulate gene expression by utilizing energy generated by ATP hydrolysis (4). The results of gene sequencing illustrated that mutations in the SWI/SNF complex accounted for approximately 20% of mutations in cancers, making it the most frequently mutated tumor suppressor in cancer (5). ARID1A is the core subunit of the SWI/SNF complex, and it can interact with DNA in a sequence-non-specific manner. ARID1A is also the most frequently mutated subunit of the SWI/SNF complex (6, 7). In the last decade, the role of ARID1A in tumors has been widely investigated, and ARID1A mutations were commonly observed in multiple cancers (5, 8). These mutations, which are primarily non-sense or frameshift mutations, can lead to the deficiency of ARID1A and further tumorigenesis (9). Recent genomic studies illustrated that ARID1A is mutated in biliary malignancies at a rate of up to 18% (10–13). Many researchers demonstrated that ARID1A inactivation is correlated with worse prognosis in patients with biliary cancer (11, 14, 15).

Recently, the tumor microenvironment (TME) has emerged as a promising target for cancer treatment, and the PD1/PD-L1 pathway plays an important role in immune evasion in TME. Some recent studies revealed the correlation between ARID1A and the PD1/PD-L1 pathway. In particular, PD-L1 expression was significantly increased in ARID1A-mutated cancers (16, 17), which could lead to impaired tumor immunity, and this might represent a potential mechanism by which ARID1A-deficient tumors escape immune surveillance.

To date, few studies have examined ARID1A mutation in patients with GBC, and its effect on the PD1/PD-L1 pathway and the prognosis of patients with GBC remains unclear. In this study, patients with GBC with low ARID1A expression had a poor prognosis, and further bioinformatics analysis illustrated that certain immune-related pathways were suppressed in ARID1A-low tumors. Immunohistochemistry (IHC) revealed that ARID1A expression was negatively correlated with PD-L1 expression and PD1+ T-cell infiltration in GBC. We propose that ARID1A inactivation could lead to a worse prognosis in patients with GBC, possibly because of impaired immune surveillance in the TME.

## Materials and Methods

### Patient Cohort

This retrospective study enrolled 244 consecutive patients with GBC who underwent surgical resection between January 2008 and December 2013 at Zhongshan Hospital, Fudan University. The diagnosis of GBC was based on preoperative imaging results, intraoperative exploration, and postoperative pathological reports. Written informed consent was obtained from all patients, and the study was approved by the ethics committee of Fudan University. The inclusion criteria were as follows: 1) age ≥ 18 years; 2) confirmation of GBC; 3) complete clinical and baseline data available; and 4) receipt of radical surgery for GBC. The exclusion criteria were as follows: 1) presence of other tumors or chronic diseases; 2) incomplete clinical or follow-up data; and 3) failure of immunohistochemical staining. Among the enrolled patients, four were excluded because of failed immunostaining, and 16 patients were excluded because of incomplete follow-up data. Finally, 224 patients with GBC were included in this cohort.

### Data Extraction

The clinicopathological and baseline characteristics of the patients, including age, gender, tumor differentiation, residual tumor, vascular invasion, and TNM stage, were collected retrospectively. The clinical staging of GBC was performed according to the eighth edition of the AJCC staging manual. Follow-up data were collected at an interval of 3 months, and overall survival (OS) was calculated from the date of surgery to that of death or the last visit.

### Tissue Microarray and Immunohistochemistry

Tissue microarray (TMA) was established in this study using formalin-fixed, paraffin-embedded surgical specimens, and the specimens were stained immunohistochemically using appropriate antibodies (anti-ARID1A [1:600, ab182560, Abcam, USA]; anti-PD-L1 [1:200, SP142, Roche, Switzerland]; anti-PD1 [1:100, ab52587, Abcam]; anti-CD8 [1:400, ab4055, Abcam]). The ARID1A staining score was calculated as the percentage of positive tumor cells (0%–100%) multiplied by the staining intensity score (0 = negative, 1 = weak, 2 = moderate, and 3 = strong). The calculation of the PD-L1 staining score followed the criteria of the IMpower110 study (18), with some changes (TC% = 0, IHC0; 0 < TC% < 1%, IHC1; 1% < TC% < 50%, IHC2; TC% ≥ 50%, IHC3). Images were acquired using a Nikon Eclipse Ti-S microscope, whereas the numbers of PD1+ tumor-infiltrating lymphocytes (TILs) per field, and CD8+ cells per field were calculated using Image-Pro Plus 6.0 software. The cutoff delineating the high and low expression subgroups was determined by the minimum p-value method using X-title software. Identical settings were applied in each photograph. 

### Gene Set Enrichment Analysis

Despite the lack of data on GBC in The Cancer Genome Atlas (TCGA), cholangiocarcinoma data were collected for differential expression analysis because the gallbladder shares the same embryonic origin as bile ducts. This is a routine workaround in the bioinformatics analysis of GBC. In total, 36 cases of cholangiocarcinoma from TCGA were analyzed, and mRNA expression data were downloaded from cBioPortal in the RSEM format. Gene Set Enrichment Analysis (GSEA) was performed to analyze the divergences of biological pathways between high and low ARID1A expression, and the differential gene expression between the high and low expression groups was explored using the edgeR package. The cutoff of ARID1A expression was determined as the median. 

### Statistical Analyses

Statistical analysis was performed using SPSS (version 25.0), Medcalc Software (version 19.0.4), Stata (version 16.0), and GraphPad Prism 8 (version 8.0.2). Patients’ baseline and clinicopathological characteristics were compared using Pearson’s chi-square test, Fisher’s exact test, and the *t*-test. OS curves were plotted using the Kaplan–Meier method, and the differences between subgroups were analyzed using the log-rank test. Independent prognostic variables were identified by Cox univariate and multivariate regression analyses. All statistical analyses were two-sided, and statistical significance was indicated by p < 0.05.

## Results

### Correlation Between ARID1A Expression and the Prognosis of Patients With Gallbladder Cancer

**Figure 1:**
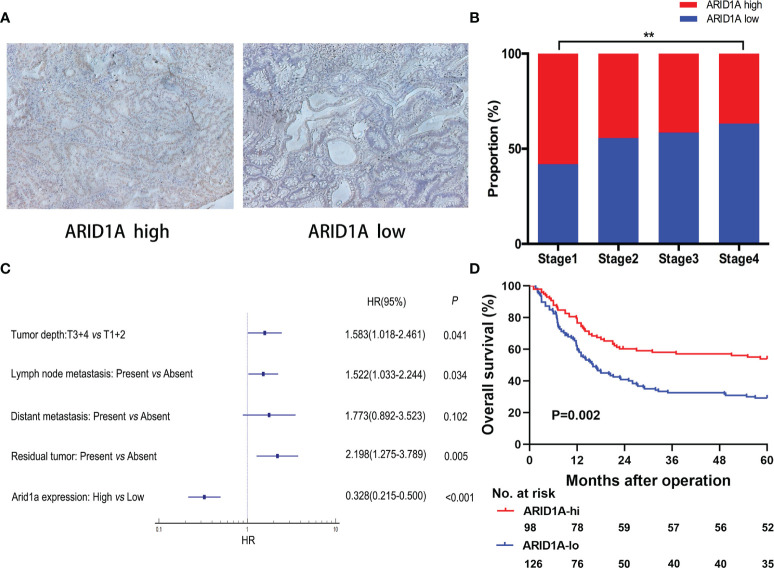
Evaluation of AT-rich interactive domain 1A (ARID1A) expression by immunohistochemical staining in patients with gallbladder cancer (GBC). **(A)** Representative immunohistochemical images of tumor tissues with high or low ARID1A expression. **(B)** Proportions of different TNM stages in patients with high or low ARID1A expression. **(C)** Multivariate Cox regression analysis identified ARID1A expression as an independent prognostic factor for overall survival. **(D)** Kaplan–Meier survival analysis of overall survival in all patients. **P < 0.01.

Immunohistochemical staining was performed using 224 GBC TMAs, and the typical images of high and low ARID1A expression are presented in **Figure 1A**. Based on the cutoff, 98 patients were included in the high ARID1A expression subgroup, and 126 patients were separated into the low ARID1A expression subgroup. The correlations between clinicopathological characteristics and ARID1A expression are presented in **Table 1**. Remarkably, lower ARID1A expression in GBC tissues was positively correlated with the TNM stage (p = 0.007, **Figure 1B**), whereas multivariate Cox regression analysis identified ARID1A expression as an independent prognostic factor (p < 0.001, **Figure 1C**). The Kaplan–Meier analysis suggested that patients with high ARID1A expression had longer OS than those with low ARID1A expression (p = 0.002, **Figure 1D**). We conclude that ARID1A expression is significantly correlated with patient prognosis in GBC, whereas high ARID1A expression predicts a better prognosis.

**Table 1:** Correlations between ARID1A expression and patient characteristics.

Characteristic	Patients (n = 224)		ARID1A expression		*p* ^a^
	Number	%	Low (n = 126)	High (n = 98)	
Age at surgery, years^b^					**0.022**
Mean ± SD	63.55 ± 11.39		62.02 ± 11.28	65.52 ± 11.29	
Gender					0.465
Female	161	71.9	93	68	
Male	63	28.1	33	30	
Tumor location					0.938
Perihilar	26	11.6	14	12	
Distal	29	12.9	17	12	
Gallbladder	169	75.5	95	74	
pT stage					0.791
T2	139	62.1	76	63	
T3	67	29.9	40	27	
T4	18	8	10	8	
pN stage					0.129
N0	181	80.8	97	84	
N1,2	43	19.2	29	14	
M stage					0.167
M0	209	93.3	115	94	
M1	15	6.7	11	4	
TNM stage					0.621
I	27	12.1	13	14	
II	100	44.6	54	46	
III	63	28.1	38	25	
IV	34	15.2	21	13	
Tumor differentiation					0.554
Well, moderate	101	45.1	59	42	
Poor	123	54.9	67	56	
Residual tumor					0.677
R0	201	89.3	114	87	
R1	23	10.7	12	11	
Vascular invasion					0.420
Absent	166	73.4	96	70	
Present	58	26.6	30	28	

ARID1A, AT-rich interactive domain 1A.

a p < 0.05 is considered statistically significant.

b The results of continuous variables are presented as the mean ± SD.

Bold values represent significant p-values (P <0.05).

### ARID1A Downregulation Correlated With T-Cell Inactivation in The Cancer Genome Atlas Dataset

**Figure 2:**
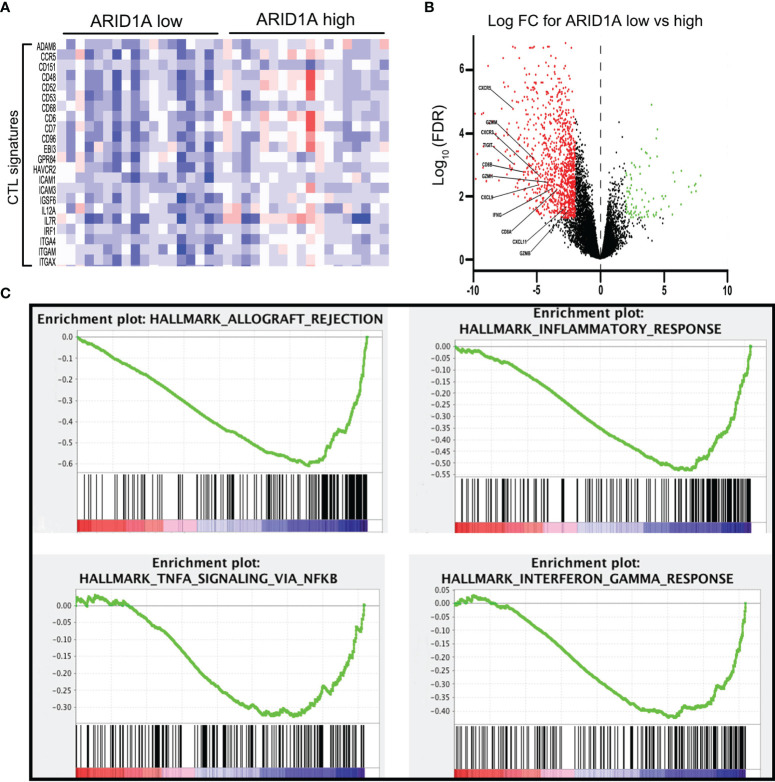
Bioinformatics analysis revealed the regulation of immune-related genes and signaling pathways in AT-rich interactive domain 1A (ARID1A)-inactivated tumors. **(A)** Heatmap of cytotoxic T lymphocyte (CTL) signatures in tumors with high or low ARID1A expression. **(B)** Volcano plot presenting the downregulation of genes involved in CD8+ T-cell activation in ARID1A-inactivated tumors. **(C)** Gene set enrichment analysis revealed the downregulation of the immune-related pathway in ARID1A-inactivated tumors.

To investigate the potential mechanism of ARID1A in GBC, we investigated gene profiles using TCGA cholangiocarcinoma cohort. As illustrated in **Figure 2A**, ARID1A expression was positively correlated with activated CD8+ T-cell signatures. To further explore the role of ARID1A, differential gene expression analyses were performed. As depicted in **Figure 2B**, genes involved in T-cell activation (e.g., GZMM, CD8A, TIGIT, and IFNG) and T-cell recruitment (e.g., CXCR3) were significantly downregulated in the low ARID1A expression group. We then performed GSEA to determine potential immune lineage changes related to ARID1A expression. Of note, multiple T-cell activation-related processes and signaling pathways were downregulated in tumors with low ARID1A expression, including allograft rejection, inflammatory response, TNF-α, and interferon-gamma (IFN-γ) signaling pathways (**Figure 2C**). These results suggest that ARID1A downregulation mediates immune evasion by impairing T-cell proliferation and activation in the biliary cancer TME. 

### Decreased ARID1A Expression Is Correlated With High PD-L1 Expression and Increased PD1+ Tumor-Infiltrating Lymphocyte Infiltration in the Gallbladder Cancer Tissue Microarray Dataset

**Figure 3:**
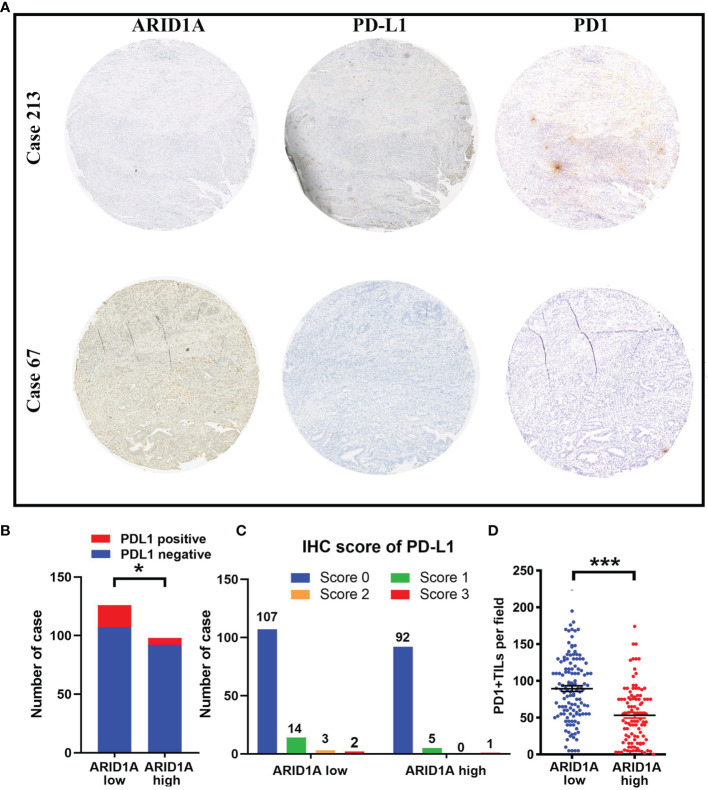
The correlation of AT-rich interactive domain 1A (ARID1A) expression with PD-L1 expression and PD1+ tumor-infiltrating lymphocyte (TIL) counts in patients with gallbladder cancer (GBC). **(A)** Representative immunohistochemical images of PD-L1 expression and PD1+ TILs in patients with low (left upper) or high (left lower) ARID1A expression. **(B)** Distribution of PD1+ TILs in different ARID1A expression groups. **(C)** Proportion of patients with positive PD-L1 staining. **(D)** Distribution of the immunohistochemistry (IHC) score for PD-L1 in the ARID1A-high and ARID1A-low groups. *P < 0.05; ***P < 0.001.

The downregulation of multiple T cell-related pathways, especially the TNF-α and IFN-γ signaling pathways, suggested the possible involvement of the PD1/PD-L1 pathway in the observed mechanism of ARID1A. Thus, we investigated the relationships of ARID1A with PD-L1 expression and PD1+ TIL counts through IHC and found that GBC tissue specimens with low ARID1A expression exhibited increased PD-L1 expression, and they were more likely to display greater PD1+ TIL infiltration (**Figure 3A**). Further, we investigated the correlation between ARID1A and PD-L1 expression, and the result revealed that the proportion of PD-L1-positive GBC tissue specimens was much higher in the ARID1A-low group than in the ARID1A-high group (p = 0.035, **Figure 3B**). Meanwhile, the proportion of GBC tissues with strongly positive PD-L1 staining on IHC (IHC score ≥ 2) was also higher in the ARID1A-low group, although the result was not statistically different (p = 0.150, **Figure 3C**). We also confirmed a negative correlation between ARID1A expression and the number of PD1+ TILs in GBC tissue (p < 0.001, **Figure 3D**).

### Low PD-L1 Expression or Reduced PD1+ Tumor-Infiltrating Lymphocyte Infiltration Combined With High ARID1A Expression Is Associated With the Best Prognosis in Gallbladder Cancer

**Figure 4:**
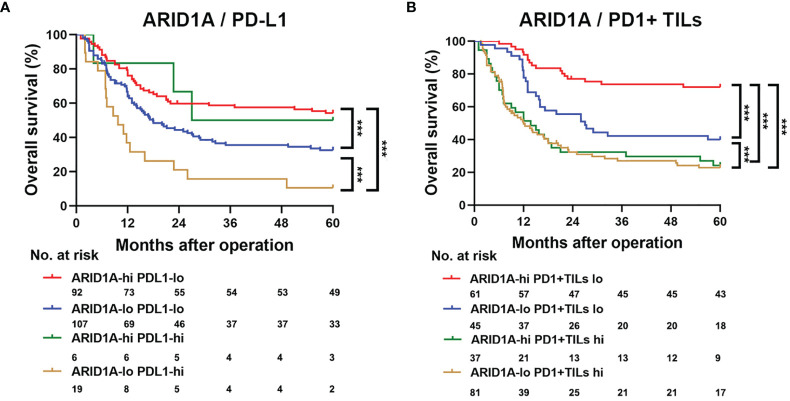
Kaplan–Meier survival analysis of overall survival (OS) according to AT-rich interactive domain 1A (ARID1A) expression, PD-L1 expression, and PD1+ tumor-infiltrating lymphocyte (TIL) counts. **(A)** Kaplan–Meier survival analysis of OS in patients with gallbladder cancer based on ARID1A expression and PD1+ TIL counts. **(B)** Kaplan–Meier survival analysis of OS based on ARID1A expression and PD-L1 expression. ***P < 0.001.

We assessed the impact of ARID1A expression, PD-L1 expression, and PD1+ TIL infiltration on the survival of patients with GBC. According to the Kaplan–Meier analysis, patients with high ARID1A expression and low PD-L1 expression in GBC tissue had the best OS (p < 0.001, **Figure 4A**). Further analysis revealed that patients with high ARID1A expression and less PD1+ TIL infiltration had markedly better OS than the other patient subgroups (p < 0.001, **Figure 4B**). 

## Discussion

The SWI/SNF complex utilizes energy from ATP hydrolysis to mobilize nucleosomes and alter the accessibility of DNA (19). Prior studies revealed that mutations in genes encoding SWI/SNF subunits were correlated with multiple malignancies (20, 21). ARID1A, which directly interacts with chromatin as a core subunit of the SWI/SNF complex, plays critical roles in cancer cell development, differentiation, and proliferation (22, 23). ARID1A is commonly classified as a tumor suppressor, and low ARID1A expression is associated with a poor prognosis in multiple cancers (24–27). However, the relationship between ARID1A expression and the prognosis of patients with GBC remains unclear. In the first part of this study, we observed that loss of ARID1A was correlated with worse OS and TNM stage in patients with GBC. Further multivariate analysis identified ARID1A as an independent prognostic factor for OS. These findings indicate that ARID1A might be a vital factor for predicting prognosis in patients with GBC.

It is known that loss of ARID1A can facilitate tumorigenesis through several mechanisms. Notably, recent studies revealed the vital effect of ARID1A on tumor immunity. One study confirmed that ARID1A plays an important role in lymphocyte development, whereas ARID1A deletion can lead to a developmental arrest in early T cells (28). Loss of ARID1A was found to have a significant correlation with the expression of IFN-γ and checkpoint genes (including PD-L1, CTLA4, and PDCD1) in microsatellite-stable colorectal cancer (29). In addition, ARID1A has been proven to serve as a biomarker for the sensitivity to immune checkpoint inhibitors (29–32).

Considering the role that ARID1A might play in tumor immunity, we examined immune-related signatures and gene sets in TCGA cholangiocarcinoma cohort. In the second part of our study, we found that ARID1A expression was correlated with multiple T cell-related processes, whereas cytotoxic T lymphocyte (CTL) signatures were significantly decreased in ARID1A-low tumors. Further differential gene expression analyses also illustrated that genes involved in T-cell activation were downregulated in ARID1A-low tumors. These findings indicate that T cells are potentially depleted in biliary tumors with low ARID1A expression. Combined with the downregulation of several related pathways (such as TNF-α and IFN-γ signaling pathways), we propose that loss of ARID1A may lead to impaired immune surveillance in the TME of biliary cancer by influencing PD1/PD-L1 pathway activity.

ARID1A shares a complex correlation with the PD1/PD-L1 pathway. Loss of ARID1A was proven to be related to high PD-L1 expression in multiple cancers, including non-small cell lung cancer (33), gastric cancer (34), ovarian cancer (35), colorectal cancer (36), and hepatocellular carcinoma (37). Patients with ARID1A-mutated tumors are more likely to benefit from anti-PD1/PD-L1 immunotherapy than patients with wild-type ARID1A tumors (38). Mechanistically, loss of ARID1A can lead to the activation of phosphatidylinositol 3-kinase/AKT signaling, which contributes to the elevated expression of PD-L1 (34, 37). Another scenario holds that ARID1A deficiency results in increased PD-L1 expression by directly removing the antagonistic effect on *Cd274* gene (which encodes PD-L1) (39). Our future goal is to confirm whether the correlation between ARID1A and the PD1/PD-L1 pathway remains valid in GBC.

In the third part of our study, immunohistochemical staining revealed significant increases in PD-L1 expression and PD1+ TIL infiltration in ARID1A-low GBC specimens. Patients with high PD-L1 expression or those with greater PD1+ TIL infiltration had a worse prognosis (**Supplementary Figure S1**). Considering these variables together, the Kaplan–Meier analysis illustrated that high ARID1A expression, low PD-L1 expression, and lower PD1+ TIL infiltration were linked to the best prognosis in GBC. Meanwhile, because it was reported that loss of ARID1A can lead to high CD8+ T-cell infiltration *via* the increased tumor mutation burden (40), we further determined the number of CD8+ T cells in GBC specimens by IHC. The results revealed no significant difference in the number of CD8+ T cells between ARID1A-high and ARID1A-low tumors (**Supplementary Figure S2**). This indicated that the increased number of PD1+ TILs in ARID1A-low GBC tumors was not attributed to an overall increase in the number of TILs, but it was potentially the result of high PD-L1 expression in tumor tissue.

**Figure 5:**
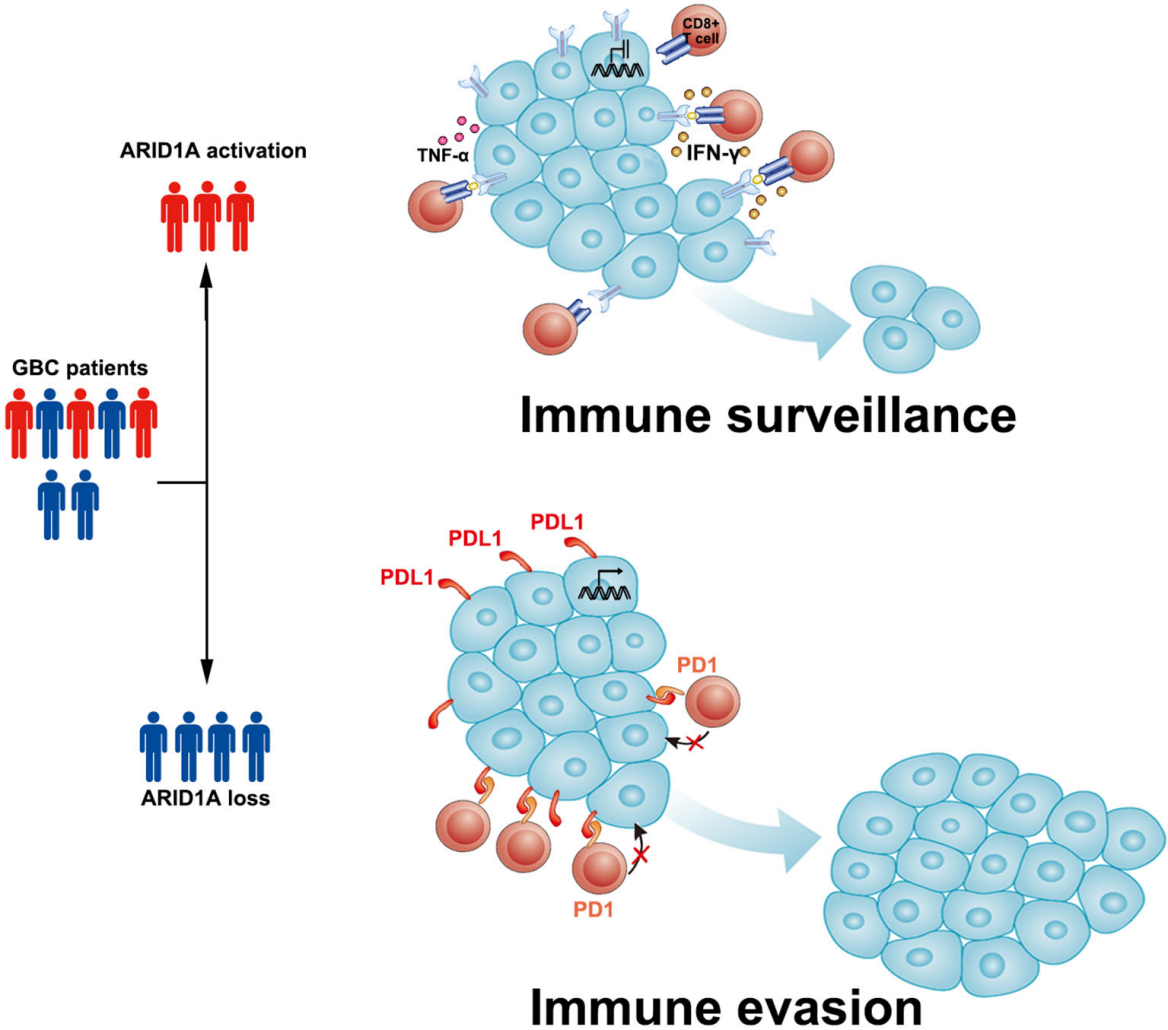
Sketch map depicting the role of AT-rich interactive domain 1A (ARID1A) in patients with gallbladder cancer (GBC). We propose that in ARID1A-activated GBC, the relatively low expression of PD-L1 allows tumor-infiltrating lymphocytes (TILs) to function normally and perform immune surveillance. In ARID1A-inactivated GBC, the upregulation of PD-L1 in tumor cells might lead to immune evasion in the tumor microenvironment *via* the PD1/PD-L1 pathway.

Based on the aforementioned findings, we propose that in GBC, ARID1A might potentially inhibit PD-L1 expression in tumor cells, which allows TILs to function normally in the TME. In ARID1A-inactivated GBC, PD-L1 overexpression and impaired TIL function lead to immune evasion by the tumor and a worse prognosis (**Figure 5**). These results suggest the potentiality of applying anti-PD1/PD-L1 therapy in patients with ARID1A-mutated GBC.

However, a few limitations should be acknowledged. As a single-center retrospective study with a relatively small sample size, it is necessary to validate these findings in a large prospective, multicenter, randomized study. In addition, the results of immunohistochemical staining in this study were based on TMA, which may not completely reflect the actual situation as a semiquantitative method. Currently, we have established an ARID1A-depleted GBC cell line, and we aim to validate these findings in humanized mice. We hope that the exact role of ARID1A in the progression of GBC will be revealed in our future work. 

## Conclusions

Our study identified the association of ARID1A downregulation with unfavorable clinical outcomes and prognosis in patients with GBC. Loss of ARID1A predicted increased PD-L1 expression and elevated PD1+ TIL infiltration, which might lead to impaired immune surveillance in the TME of GBC. The combination of high ARID1A expression, low PD-L1 expression, and reduced PD1+ TIL infiltration predicted the best OS in patients with GBC. ARID1A is a prognostic factor, and it might serve as a marker to predict the efficacy of immunotherapy. 

## Data Availability Statement

The raw data supporting the conclusions of this article will be made available by the authors, without undue reservation. 

## Ethics Statement

The studies involving human participants were reviewed and approved by the ethics committee of Fudan University (approval no.: B2018-159R). The patients/participants provided their written informed consent to participate in this study. Written informed consent was obtained from the individual(s) for the publication of any potentially identifiable images or data included in this article. 

## Author Contributions

All authors contributed to the article and approved the submitted version. LN and CW: acquisition of data, analysis, and interpretation of data, statistical analysis, and drafting of the manuscript. JW and SZ: technical and material support. XB, YW, and HL: study concept and design, analysis and interpretation of data, drafting of the manuscript, obtained funding, and study supervision. All authors contributed to the article and approved the submitted version.

## Funding

This study was supported by grants from the National Natural Science Foundation of China (81872352, 82002525), the Foundation of Shanghai Science and Technology Committee (20JC1418902), the Shanghai Sailing Program (20YF1407300), the Science and Technology Commission of Shanghai Municipality (20DZ2254500), the Clinical Research Project and Outstanding Backbone Scheme of Zhongshan Hospital (2020ZSLC55, 2021ZSGG17), and the Scientific Research Project of Shanghai Municipal Health Commission (201940389).

## Conflict of Interest

The authors declare that the research was conducted in the absence of any commercial or financial relationships that could be construed as a potential conflict of interest.

## Publisher’s Note

All claims expressed in this article are solely those of the authors and do not necessarily represent those of their affiliated organizations, or those of the publisher, the editors and the reviewers. Any product that may be evaluated in this article, or claim that may be made by its manufacturer, is not guaranteed or endorsed by the publisher.

## Supplementary Material

Supplementary Figure S1 Effects of PD-L1 expression and PD1+ tumor infiltrating lymphocytes (TILs) on overall survival (OS) in patients with gallbladder cancer. **(A)** The OS curves of patients with high or low PD-L1 expression. **(B)** The OS curves of patients with high or low PD1+ TIL infiltration.

Supplementary Figure S2 The comparison of CD8+ cells and PD1+ tumor infiltrating lymphocytes (TILs) in samples with high/low expression of ARID1A.
