# Supplementary figures and images for: ARID1A Downregulation Predicts High PD-L1 Expression and Worse Clinical Outcome in Patients With Gallbladder Cancer
Source: Front Oncol. 2022 Feb 7;12:787897. doi: 10.3389/fonc.2022.787897 (PMC8858979; doi:10.3389/fonc.2022.787897)

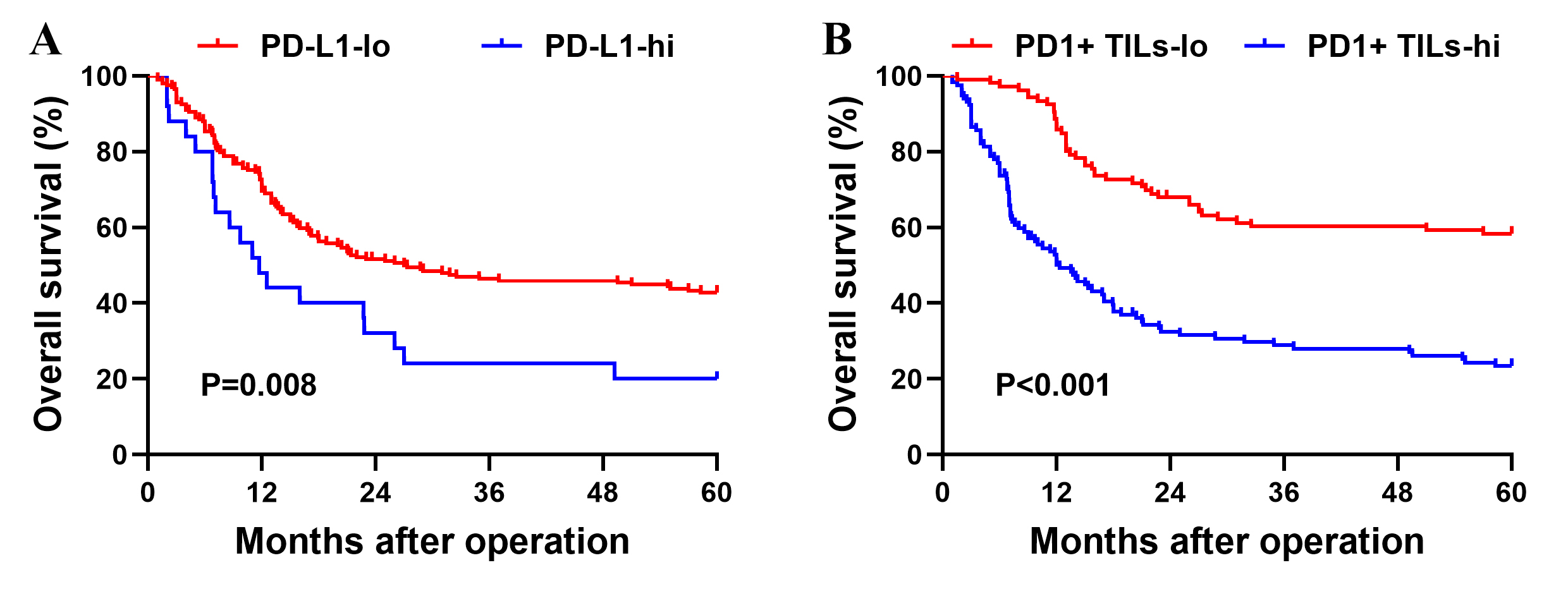

Supplement: Supplementary Figure S1 — Effects of PD-L1 expression and PD1+ tumor infiltrating lymphocytes (TILs) on overall survival (OS) in patients with gallbladder cancer. (A) The OS curves of patients with high or low PD-L1 expression. (B) The OS curves of patients with high or low PD1+ TIL infiltration. [file Image_1.jpeg]

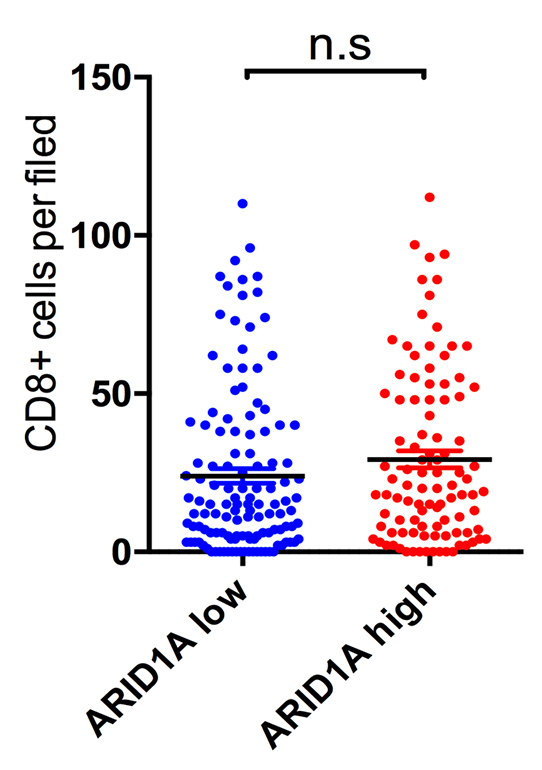

Supplement: Supplementary Figure S2 — The comparison of CD8+ cells and PD1+ tumor infiltrating lymphocytes (TILs) in samples with high/low expression of ARID1A. [file Image_2.jpeg]
